# Trends and duration of antibacterial drug supply chain issues in the United States, January 2017–June 2022

**DOI:** 10.1017/ash.2023.382

**Published:** 2023-09-29

**Authors:** Katie Suda, Katherine Callaway Kim, Inma Hernandez, Mina Tadrous

## Abstract

**Background:** Drug manufacturing and distribution is a complex, global process. The global drug supply chain is prone to disruptions associated with geopolitical issues, trade, civil unrest, severe weather, and pandemics, all of which have the potential to affect medication supply and result in drug shortages. To our knowledge, the extent to which the supply of antimicrobials is threated due to disruptions in the drug supply chain in the United States is unknown. We examined trends and duration of disruptions to the drug supply chain for antimicrobials. **Methods:** Manufacturer reports of supply disruptions were extracted from the Food and Drug Administration (FDA) and the American Society for Health-Systems Pharmacists (ASHP) websites and merged on the agent-formulation level. For each month of the study period, a drug was considered to have an active supply chain issue if an FDA or ASHP shortage or recall report overlapped with that month for ≥15 days, or if a discontinuation had occurred within the previous 3 months. Total months of supply chain issues were summed for antimicrobials overall, at the agent formulation , and class levels. A Mann-Kendall test was used to determine the significance of trends in supply-chain issues. **Results:** Of 105 antimicrobials purchased in the United States, 74 (70%) had a supply-chain issue for ≥1 month from January 15, 2017, to June 30, 2022. Combined, the 74 agents had 1,611 total months of supply-chain issues over the 66-month study period. Agents from the penicillin class were most frequently affected (ie, 80% of penicillins had supply-chain issues for 206 months), but cephalosporins had supply-chain issues for the longest duration (66% of cephalosporins for 653 months). From 2017–2021, supply-chain issues decreased significantly for penicillins and quinolones (tests of trend, *P* = .01 and .02, respectively). No trend was identified for the other classes or antimicrobials overall. Interestingly, supply-chain issues for most classes did not increase with seasonal increases in antimicrobial use. Also, supply-chain issues affected 33 antimicrobial agents for at least half of the study period, and supply-chain issues affected ampicillin-sulbactam, cefotaxime, ceftazidime, cefotetan, cefepime, clindamycin, vancomycin for 100% of the study period. **Conclusions:** Drug supply-chain issues commonly affect antimicrobials and are not improving for most classes. Drug supply-chain issues cause significant strain on healthcare, including drug procurement, access to optimal therapy, and poses challenges to prescribing and antimicrobial stewardship. To decrease the threat to the antibacterial drug supply, action should be taken to strengthen the drug supply chain to ensure access to these essential medicines.

**Disclosures:** None

